# Finding the Value: Identifying the Key Elements of Recorded Clinic Visits From the Perspective of Patients, Clinicians, and Caregivers

**DOI:** 10.1111/hex.70143

**Published:** 2025-01-08

**Authors:** Paul J. Barr, Garrett T. Wasp, Michelle D. Dannenberg, Lisa A. Mistler, Kanak Verma, Kyra Bonasia, William R. Haslett, Craig H. Ganoe, Reed W. Bratches, Karen Schifferdecker

**Affiliations:** ^1^ The Dartmouth Institute for Health Policy & Clinical Practice, Dartmouth College Lebanon New Hampshire USA; ^2^ Center for Technology and Behavioral Health, Dartmouth College Lebanon New Hampshire USA; ^3^ Department of Internal Medicine Dartmouth‐Hitchcock Medical Center Lebanon New Hampshire USA; ^4^ Dartmouth Cancer Center at Dartmouth Health Lebanon New Hampshire USA; ^5^ Geisel School of Medicine at Dartmouth College Hanover New Hampshire USA; ^6^ School of Nursing, MGH Institute of Health Professions Boston Massachusetts USA; ^7^ Stanford Health Care, Stanford Medical Center Stanford California USA; ^8^ Department of Obstetrics and Gynecology University of Ottawa Ottawa Canada; ^9^ Department of Biomedical Data Science Dartmouth College Hanover New Hampshire USA; ^10^ University of Alabama at Birmingham School of Nursing Birmingham Alabama USA

## Abstract

**Objective:**

We aimed to understand what patients, caregivers and clinicians identified as the most important information from their audio‐recorded clinic visits and why.

**Methods:**

We recruited patients, caregivers and clinicians from primary and speciality care clinics at an academic medical centre in New Hampshire, U.S. Participants reviewed a recording or transcript of their visit, identifying meaningful moments and the reasons why. Two researchers performed a summative content analysis of the data.

**Results:**

Sixteen patients, four with caregivers, from six clinicians participated. Patients, caregivers and clinicians identified a median of 7.5 (3–20), 12.5 (6–50) and 18 (4–31) meaningful visit moments, respectively. Moments identified were similar across stakeholders, including patient education, symptoms, recommendations and medications. Four themes emerged as a rationale for finding visit information meaningful: providing and receiving information, sharing the patient experience, forming a care plan, and providing emotional support. Clinicians rarely identified patient statements as important.

**Conclusion:**

There was considerable agreement between patients, clinicians and caregivers regarding visit information that is most valuable. Patient contributions may be undervalued by clinicians.

**Practice Implications:**

These findings can be used to improve patient‐centred visit communication by focusing visit summaries and decision support on information of the most value to participants.

## 1 Introduction

Consulting with a clinician continues to be the patient's most trusted resource for medical information [1, 2]. Patients and their family caregivers rely on verbal communication with their clinicians to make healthcare decisions and to learn how to manage their illnesses [3]. Current models of healthcare delivery, such as the coproduction model of care, focus on improving the quality of information exchanged before, during and after the clinic visit. In the coproduction model of care, patients bring their perceptions of health and function to the visit, sometimes via patient‐reported outcome measures (PROMS) [4], while the clinician contributes medical information supported by clinical data and their experience [5]. Taken together, both parties review this data in real time to reach a shared decision on the best path forward. Post‐visit, patients have access to after‐visit summaries [6] or their patient portal to review recommendations from the visit, often with the support of a family member or friend [7]. This information can then be used to support self‐management, increase confidence in family caregivers to support patients and prepare for future visits.

Unfortunately, the ideal version of coproduction of care is not the norm. For example, patients report that after‐visit summaries contain inaccurate information, are complex, and often have errors [8, 9], while levels of shared decision making in visits are often low [10]. Contributing to this lack of coproduction is a gap in our understanding of what information exchanged during the visit is most valued by patients and why. Identifying what is most important to patients in the visit encounter can promote better shared decision making, while providing information that patients desire during a medical visit is associated with better outcomes, such as improved satisfaction, less depression or anxiety, better medical decision‐making and improved physiologic outcomes [11, 12].

In a systematic review of health information needs among primary care patients, the most desired information by patients was information on their disease, followed by nutrition and then available treatments [13]. Studies in the review use interviews, focus groups, questionnaires, and computer logs of the electronic health record (EHR) to identify information needs [13]. Yet a limitation of prior methods is that patients are asked to reflect hypothetically on what information they may want from their clinic visits, and information documented in the EHR log, noted by the clinician, may not capture what information is of most value to patients.

An under‐explored strategy to identify visit information of most value to patients, clinicians and caregivers is to review the audio recording of their clinic visits. Triangulating the perspectives of these stakeholders could offer important insights to inform the design of tools and strategies to support patient‐centred communication. Knowing what is of value to patients can guide clinicians in framing messages and eliciting motivation, essentially tailoring their communication strategies to each individual. In this exploratory project, we aimed to identify and compare what patients, clinicians, and caregivers feel is the most important information from a clinic visit and the reason why.

## 2 Materials and Methods

### 2.1 Study Design

We conducted a qualitative study of primary and speciality care clinicians, their patients, and caregivers of patients (when present) to identify what information from a clinic visit was important and why. This study received IRB approval from the Committee for the Protection of Human Subjects (CPHS) at Dartmouth College (STUDY# 29708). This study is reported in accordance with the ‘Consolidated Criteria for Reporting Qualitative Research’ (COREQ) guideline [14].

### 2.2 Setting & Participants

We recruited clinicians, patients, and caregivers from four specialty clinics (orthopaedics, cardiology, radiation oncology, and gynaecologic oncology) within a large academic medical centre and an affiliated outpatient primary care clinic in the Upper Valley of New Hampshire between November 2016 and June 2018. Participants had to be at least 18 years of age and able to communicate in English. Participants who could not provide informed consent or had hearing impairment were excluded from the study. Convenience samples of clinicians were recruited via email. Recruited clinicians were asked to identify patients who would be receiving a new health‐related diagnosis or making a medical decision during their visit (i.e., new treatment, screening decision), rather than those coming in strictly for a procedural visit (i.e., blood draw or immunization) where little information is exchanged. If caregivers were present and the patient agreed to their participation, they were invited to complete informed consent and participate in the project. We aimed to recruit a minimum of six to eight patients from both primary care and specialty care, reflecting a number of interviews considered adequate to reach data saturation [15].

### 2.3 Study Procedures

**Figure 1:**
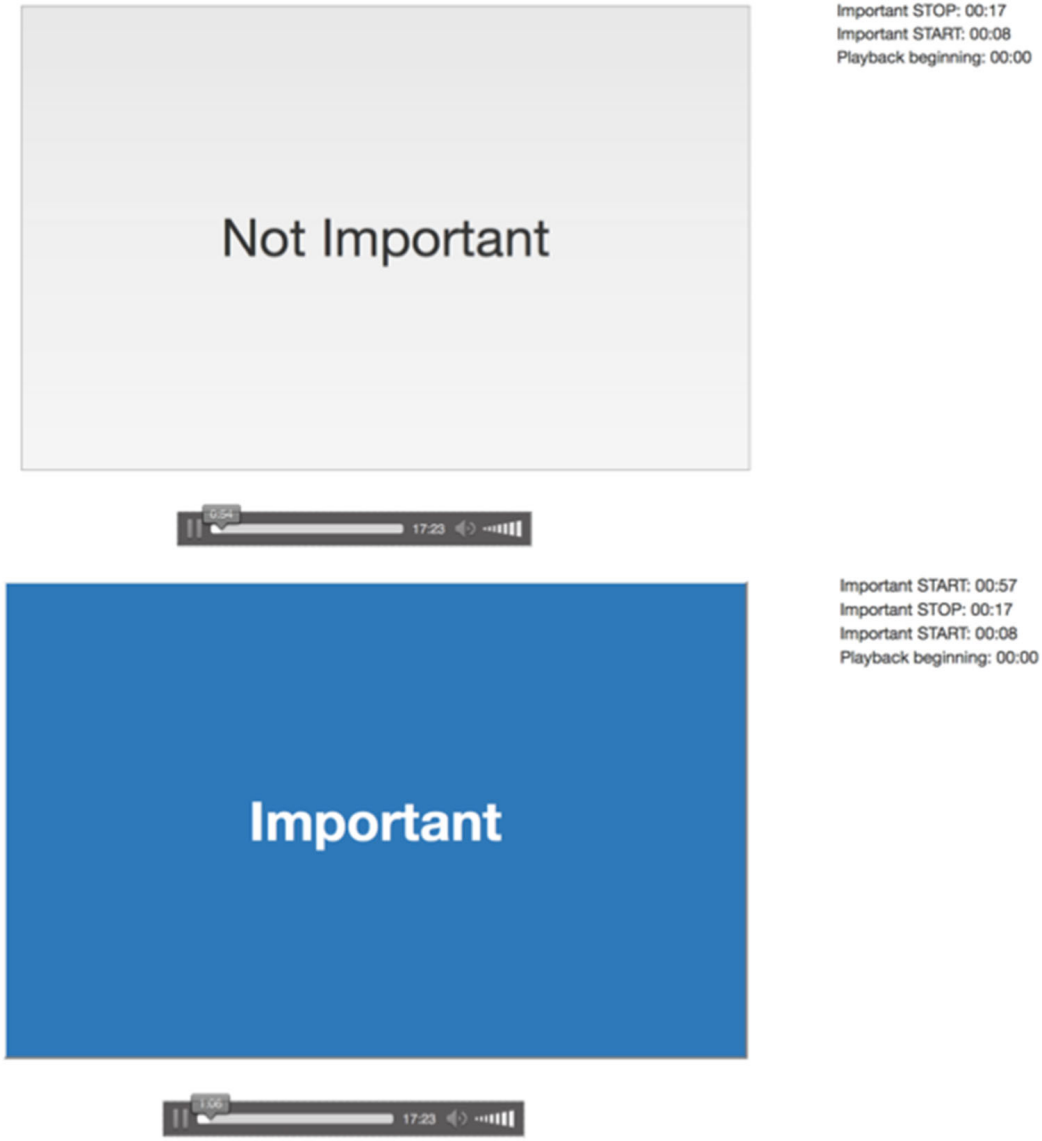
Software capturing a participant indicating hearing something important in their visit recording.

Patients received a portable digital audio recorder and were instructed to begin recording at the start of their clinic visit and stop the recording upon the visit conclusion. Immediately after the clinic visit, patients were brought into a separate clinic room by a session facilitator. Patients were instructed to listen back to the clinic visit and identify (1) important information that could include something they may want to recall later or want a family member to hear, and (2) why the segment was important to them. Patients used a secure application developed by researchers at Dartmouth College to play back the recording and identify the parts of the visit that were most important. To highlight important information, patients would press and hold the spacebar, releasing the spacebar when the important information concluded (Figure 1). Patients' responses to why the segment was important were written down verbatim by the facilitator. Caregivers completed the same study procedures independently. Both received $20 for their participation. Due to time constraints, audio recordings from clinic visits were transcribed verbatim by Acusis, a HIPAA‐compliant medical transcription company, and shared with clinicians. Clinicians were instructed (1) to mark on the transcript information from the visit they, as clinicians felt, was most important and what they would want the patient to remember, and (2) to provide their rationale for each section of the text identified. Clinicians were not paid for their participation in the study.

### 2.4 Data Collection

We collected patient and caregiver demographics (age, gender, race, ethnicity, education), recordings of clinic visits, written and verbal responses on what information from the recordings or transcripts was important, and reasons information was deemed important.

### 2.5 Data Analysis

We conducted a summative content analysis, counting and comparing the utterances considered most valuable by stakeholders, to identify what themes of information from the clinic visit were important [16]. Our approach to coding was informed by findings from our case study of clinics where audio/video recordings of clinic visits are routinely shared with patients and caregivers; in the case study, interviewed stakeholders stated what information was most valuable from recordings, but they did not review and identify information [17]. We also conducted a directed content analysis to identify themes for why the information was identified as important. Two coders independently applied an initial codebook to a subset of the clinician rationale data. The two coders then met to create a revised codebook, which was applied to the existing codes. Emergent codes were added to existing codes. This process was repeated for the patient and caregiver rationale data. The research team decided to create separate codebooks for each stakeholder group to capture nuanced differences in the codes that emerged between groups. Codes were reviewed to create qualitative memos which allowed the researchers to interpret the data and identify emerging themes. Memos were reviewed to search for themes across stakeholders. Potential themes were reviewed and refined with a third reviewer. To increase trustworthiness and credibility, qualitative study results were reviewed independently by a methods expert who was not part of the core research team (KS). Statements made exclusively by the clinician were classified as the clinician speaking, statements made exclusively by the patient were classified as the patient speaking, and statements where both the patient and clinician spoke/contributed to the same topic (i.e., dialogue) were classified as patient and clinician speaking. All quotations presented in the results section have been deidentified.

## 3 Results

### 3.1 Participant Characteristics

Seventeen unique clinic visits, including eight primary care and nine specialty care visits, were recorded and included in the analysis (Table 1). Six clinicians reviewed a median of three visit recordings, as they were the treating clinicians for more than one patient in the sample. A total of 16 patients and 4 caregivers reviewed their visit recordings. One primary care clinician was unable to review three of their visit recordings due to illness and one patient did not review their visit recording. Patients had a median age of 68 years (25–84 years), more than half were female (56%) and more than half (63%) possessed a bachelor's degree or higher (Table 2). Caregivers had a median age of 57 years (46–78 years), were predominantly female (75%), and had a graduate degree or higher (75%). Four out of the six clinicians were male, and all had been in practice for at least 10 years, with three having practised for over 25 years. Clinician specialties included family medicine (*n* = 2), interventional cardiology, gynaecologic oncology, radiation oncology, and orthopaedic surgery.

**Table 1:** Participant breakdown by visit (17 unique patient visits recorded).

Visit	Type	Reviewed		
		Patient	Clinician	Caregiver
1	Primary Care	x	x	—
2	Primary Care	x	x	—
3	Primary Care	x	x	—
4	Primary Care	x	x	—
5	Primary Care	x	x	—
6	Primary Care	x	—	—
7	Primary Care	x	—	x
8	Primary Care	x	—	—
9	Orthopaedic Surgery	x	x	—
10	Gynecologic Oncology	x	x	—
11	Radiation Oncology	x	x	x
12	Radiation Oncology	x	x	x
13	Orthopaedic Surgery	x	x	x
14	Gynecologic Oncology	x	x	—
15	Radiation Oncology	x	x	—
16	Cardiology	x	x	—
17	Cardiology	—	x	—

**Table 2:** Patient (*n* = 16) and caregiver characteristics (*N* = 4).

	Patients (*n* = 16)^a^	Caregivers (*n* = 4)
**Age (years),** median [range]	68 [25–84]	57 [46–78]
**Gender (female), *n* (%)**	9 (56%)	3 (75%)
**Race** ***(White)***, ***n* (%)**	16 (100%)	4 (100%)
**Ethnicity** * ***(Non‐Hispanic)***, ***n* (%)**	15 (94%)	4 (100%)
**Education, *n* (%)**		
*High school or less*	5 (31%)	—
*Some college*	1 (7%)	1 (25%)
*Bachelors*	5 (31%)	—
*Master's or higher*	5 (31%)	3 (75%)

a Not provided by one participant.

The average length of visits was 33:43 min [8:10–61:05]. The median number of total words exchanged during a visit was 5271.5 [1466–8971], of which 37% were spoken by patients [7%–60%], 59% by clinicians [40%–92%], and 4% by others [1%–12%].

### 3.2 Information Identified As Important

A total of 440 statements were identified as important by stakeholders across the visits (Table 3). Clinicians identified a median of 12.5 statements [range 6–50 statements] while patients identified a median of 7.5 statements [range 3–20 statements], a ratio of 1.6 statements identified by clinicians for every 1 statement identified by patients. Caregivers identified a median of 18 statements [range 4–31 statements] per visit reviewed. Overall, information related to patient education was most frequently identified as important (38.6%; 170 statements). This included background information on a medical condition and explaining how a medical procedure works (see Table 3). Education was followed by symptoms and problems (18%; 78 statements), the clinician's recommendation (18%; 77 statements), and the discussion of medications (15%; 64 statements). When doctors identified information related to medications as important, it tended to focus on side effects, whereas patients also identified information about medication benefits as important.

**Table 3:** Classification of statements identified as important by stakeholders.

	Clinicians	Patients	Caregivers^a^	Total
**Unique visits recorded (*N*** = **17)**				
Visits reviewed by stakeholder (N)	14	16	4	
Statements identified as Important (N)	227	138	39	440
Median statements identified per visit	12.5	7.5	18	
**Statement topic classifications % (*N*)**				
Education	44% (101)	38% (52)	44% (17)	39% (170)
Signs, Symptoms, and Problems	15% (33)	26% (36)	23% (9)	18% (78)
Recommendation	24% (55)	13% (18)	10% (4)	18% (77)
Discussion of Medications	13% (30)	21% (29)	13% (5)	15% (64)
Test and imaging results	7% (17)	14% (19)	10% (4)	9% (40)
Follow‐up Appointments	10% (23)	9% (13)	3% (1)	8% (37)
Treatment options	7% (17)	8% (11)	10% (4)	7% (32)
Diagnosis	1% (3)	1% (1)	—	1% (4)
Healthcare experience	1% (3)	—	—	1% (3)
Clinician/Team Role	< 1% (1)	1% (1)	—	1% (3)
Physical exam	—	1% (1)	—	< 1% (2)

a Caregivers were present at 4 of 17 patient visits. Additionally due to a software error that failed to capture the visit timestamp on some statements during three visits only 39 statements were evaluable.

Clinicians overwhelmingly selected their own statements as important (85.5% of statements). Caregivers also identified primarily information conveyed by the clinician as important (92.3% of statements). Patients were equally likely to identify the information they shared or their dialogue with the clinician as important (48.6% of statements), compared to the clinicians' statements alone (51.4% of statements). None of the statements that were identified as important was spoken by a caregiver (Table 4).

**Table 4:** Important statement speaker frequency and examples.

	Clinician	Patient	Caregiver^a^
Total statements identified by stakeholder	227	138	39
**Statement source/speaker % (*N*)**			
Clinician speaking	85.5% (194)	51.4% (71)	92.3% (36)
Patient speaking	7.0% (16)	13.8% (19)	2.6% (1)
Clinician and patient speaking	7.5% (17)	34.8% (48)	5.1% (2)

a For Caregiver, due to a software error that failed to capture the visit timestamp on some statements during three visits only 39 statements were evaluable.

### 3.3 Thematic Analysis of Why Information Is Important

Four main themes emerged for why the information was identified as important: knowledge transfer on key information, the patient experience, the care plan and the provision of support/reassurance by the clinician.

#### 3.3.1 Theme 1: Providing and Receiving Information

The provision of education about the condition was the most common reason provided by participants for highlighting a section of the visit as valuable. Providing education was often unidirectional (i.e., clinician providing patient information about disease, resources and treatments). Sometimes bidirectional learning was cited as important (shared learning that contextualized the medical information). For clinicians, providing information to educate patients on their medical issue(s) was the most common rationale for identifying information as important. Educating patients on their medical issue(s) ranged from providing a ‘big picture’ overview (i.e., explaining a condition) to reviewing test results and explaining why a medical course of action is being pursued. This theme included the use of education as a means of persuasion, for example, ‘*introducing the idea of trying some chiropractic care for this patient who has chronic vague musculoskeletal pain everywhere*’ (CL01). From the patient's perspective, correspondingly receiving information related to their disease, medical treatment and testing was the most common reason for patients to identify information as important. Patients felt that this information helped them understand and act on the clinician's advice and increased awareness of options. The notion of patient choice was also echoed within the clinician's rationale; for example, ‘*education about relationships between risk and need for Pap [smear] ‐ patient knowledge about her choice*’ (CL01). From the clinician's perspective, the communication of educational content was enhanced by checking in with the patient to align comprehension. This included reasons such as ‘*ensure patient understanding of script to avoid confusion later*’ (CL05) and ‘*checking for comprehension of plan*’ (CL06). From the patients' perspective, they valued the clinician summarizing information, for example, one patient mentioned the ‘*clinician sums up all tests needed*… *good summary sendoff*’ (P03).

#### 3.3.2 Theme 2: Sharing the Patient Experience

For patients, it was important to share information to help their clinician (1) understand historical information about their condition and family history, (2) explain how they are currently doing, (3) express concerns about their lifestyle or different treatment options and (4) explain what they are doing for their health. For caregivers, the patient sharing how they are doing and the symptoms they experienced was important; for example, ‘*significant arthritis symptoms to the rest of the body*’ (CG03). Clinicians indicated that the patients' descriptions of how their health issue or healthcare experience impacted them were important; for example, ‘*good insight from the patient herself that her chronic pain feeds into her depression*’ (CL01). However, clinicians cited these statements as important to a lesser extent than either patients or caregivers did.

#### 3.3.3 Theme 3: Forming a Care Plan

Actionable information provided by clinicians as a recommendation was important to all stakeholders. For clinicians, this information was mostly prescriptive (i.e., instructions for the patient), although sometimes space was left for modification; for example ‘*recommending a plan but checking with the patient*’ (CL06). Patients and caregivers also commonly cited care planning, such as *‘referral to primary care’* (P12) and *‘Future appt [appointment] plan to check Vitamin D level at next visit’* (P06) as their rationale for identifying information as important, although to a lesser extent than clinicians.

#### 3.3.4 Theme 4: Providing Emotional Support

Clinicians felt that it was important to acknowledge concerns, reassure the patient and demonstrate they ‘*heard’* the patient. One example stated rationale of this is ‘*letting patient express concerns, symptom, feelings (just listening)’* (CL06). Correspondingly, patients noticed these supportive comments and also cited them as important. Patients cited reassurance as important, ‘*HAPPY NEWS – positive results and doctor provided a satisfactory explanation of disease prognosis’* (P13) and supportive talk ‘*Patient feedback and doctor acknowledging suggestion; instills deeper trust in doctor's treatment plan since she understood concerns’* (P16).

## 4 Discussion and Conclusion

### 4.1 Discussion

#### 4.1.1 Main Findings

In this exploratory study, we asked patients, caregivers and clinicians to review their visit interaction audio and identify which parts of the visit were of most value and why. Participants cited patient education, symptoms and problems, clinician recommendations, and medications most frequently as important. Participants indicated that visit content was important for one of four main reasons: providing and receiving information, discussing patient experience, forming a care plan, and providing emotional support. Across all three stakeholders, information exchange often in the form of education was the most common reason for identifying information as important. While discussing the patient experience was the second most common reason for information being important to patients and caregivers, the second most common reason for clinicians was forming a care plan. Compared to clinician and caregiver participants who most frequently identified statements made exclusively by the clinician as important, patients were much more likely to cite their own contributions, or the dialogue between them and their clinicians, as important; in addition, patients on average identified the fewest number of statements per visit.

#### 4.1.2 Relevance to Existing Literature

While exploratory, our finding that patients cite education most frequently as important supports prior work: the majority of a clinic visit, regardless of specialty or duration of visit, is spent on patient education [2, 18, 19]. In addition, similar to our project, prior research has found that patients desire information on their illness, treatment, care plan and side effects [2, 13]. According to Deledda et al. [20] in most quantitative, survey‐based studies, patients reported information exchange as most important while in qualitative studies, patients identified ‘fostering the relationship’ as of value. While there was consensus on information exchange, differences emerged on what information was of the most value. In contrast to clinicians who cited *recommendations*, patients and caregivers more frequently identified content on signs, symptoms, problems and medications as important. This aligns with findings from a pilot study assessing primary care clinician information needs to inform ambulatory visit note display [21]. Through semi‐structured interviews, clinicians highlighted the history of present illness and assessment & plan to be the most important sections of a visit note for primary care clinicians [21]. In our study, clinicians contributed much more to the clinic visit than patients (58% of words spoken). This is higher than reported in prior research in primary care settings where clinicians and patients contributed equally to the visit, and may be due to the inclusion of other clinical specialties (e.g., orthopaedics, cardiology, radiation oncology, and gynecologic oncology) in addition to primary care in our sample.

While our sample of caregivers was small, findings mostly support prior work that caregivers and patients want similar information from the visit. Our caregivers highlighted much more visit information as important compared to patients. Prior studies have found that caregivers often want more treatment information than patients, and in our study the breakdown of statements considered of most value more closely resembled clinicians [22, 23]. Interestingly, in this sample, no statements made by caregivers were identified as important. While this is too small a sample from which to draw conclusions, caregivers often report feeling less included in office visits [24]. This may be reflected in our study by the lack of reported meaningful contributions. This is concerning as caregivers often have valuable insights into the patient care experience; however, the addition of caregivers creates a triadic model of communication which introduces additional complexities.

#### 4.1.3 Strengths and Limitations

Our findings must be interpreted in light of several limitations. Our sample size is small, and the study population was white and highly educated, which limits generalizability. To reduce the data collection burden on clinicians we had them review written transcripts days after the visit whereas patients and caregivers reviewed audio‐recordings immediately after the visit. While we do not think these different methods and timing would cause participants to select different content or provide different rationales, we may have introduced a potential bias by using two different approaches. A strength of our project is that the findings are based on the participants' review of their actual office visit and not a survey or focus group about the information they feel would be most important, which heightens the ecological validity of our analysis. A review by Deledda et. al. [20] concluded that the way communication studies are conducted affects results. By having each stakeholder directly review their actual visit recording, we infer this helped to both minimize recall bias and ground their perspective in the actual visit rather than hypothetical information needs.

### 4.2 Practice Implications

In this exploratory study, both clinicians and caregivers placed more value on clinician statements, compared to patients who also identified their own contributions as of high value. The implications for how we communicate with patients are important—for example, an after‐visit summary could benefit from a more detailed description of what the patient contributed to the clinic visit, their thoughts, concerns and questions. These implications align with views from experts in the field of shared decision‐making who report that it is insufficient to focus on ‘*dispassionate and objective evidence of medical science*’ alone when communicating with patients [25]. It is also necessary to understand the patient's values and what matters most to each patient. By focusing on medically oriented goals, clinicians may inadvertently miss or not provide enough attention to information considered valuable to patients; this can also result in contextualized errors, ‘*the failure to adapt research evidence to patient context, when it results in an inappropriate plan of care’* [26]. In a study of 4496 clinic visits at the VA, clinicians addressed such patient contextual factors 67% of the time, which improved to 72% after clinicians were more systematically made aware of these contextual errors, which improved patient outcomes and saved an estimated $25.2 million from avoided hospitalizations [26].

### 4.3 Conclusion

Our conclusions regarding all stakeholders can only be preliminary, given the limited number of participants, particularly clinicians and caregivers. That being said, all stakeholders in our study found the exchange of information, especially patient education and care planning, to be valuable. There was overlap in what stakeholders identified as important; however, notable differences exist, such as the patients' focus on their own contributions to the visit, which was infrequently cited by clinicians. Additionally, clinicians, as reported elsewhere, dominate what is said. While this is important, more must be done to recognize the importance that patients place in their own words. The patient voice is the raw data that drives medicine; we must value it more to achieve the true coproduction of care.

## Author Contributions

**Paul J. Barr:** conceptualization, investigation, methodology, validation, supervision, writing–original draft, visualization. **Garrett T. Wasp:** formal analysis, visualization, project administration, writing–original draft. **Michelle D. Dannenberg:** conceptualization, investigation, formal analysis, data curation. **Lisa A. Mistler:** writing–review and editing. **Kanak Verma:** writing–review and editing, data curation. **Kyra Bonasia:** data curation, writing–review and editing. **William R. Haslett:** software, writing–review and editing. **Craig H. Ganoe:** software, writing–review and editing. **Reed W. Bratches:** writing–review and editing. **Karen Schifferdecker:** methodology, validation, writing–review and editing.

## Disclosure

We would like to acknowledge the guidance of Roger Arend and Sheri Piper, patient partners who have supported our research team by joining weekly meetings giving guidance on the project design, and preparation of project materials.

## Ethics Statement

Institutional Review Board (IRB) approval for the research was reviewed locally by the Committee for the Protection of Human Subjects (CPHS) at Dartmouth College (STUDY# 29708). This study received IRB approval from the Committee for the Protection of Human Subjects (CPHS) at Dartmouth College (STUDY# 29708).

## Consent

All participants consented to participation prior to before any study activities.

## Conflicts of Interest

The authors declare no conflicts of interest.

## Data Availability

Data contains patient health information and is not available for distribution.

## References

[1] B. W. Hesse , D. E. Nelson , G. L. Kreps , et al., “Trust and Sources of Health Information,” Archives of Internal Medicine 165, no. 22 (2005): 2618, 10.1001/archinte.165.22.2618. 16344419

[2] I. Ramsey , N. Corsini , M. D. J. Peters , and M. Eckert , “A Rapid Review of Consumer Health Information Needs and Preferences,” Patient Education and Counseling 100, no. 9 (2017): 1634–1642, 10.1016/j.pec.2017.04.005. 28442155

[3] D. M. McCarthy , K. R. Waite , L. M. Curtis , K. G. Engel , D. W. Baker , and M. S. Wolf , “What Did the Doctor Say? Health Literacy and Recall of Medical Instructions,” Medical Care 50, no. 4 (2012): 277–282, 10.1097/MLR.0B013E318241E8E1. 22411440 PMC3305916

[4] K. P. Weinfurt and B. B. Reeve , “Patient‐Reported Outcome Measures in Clinical Research,” Journal of the American Medical Association 328, no. 5 (2022): 472–473, 10.1001/JAMA.2022.11238. 35838745

[5] M. Batalden , P. Batalden , P. Margolis , et al., “Coproduction of Healthcare Service,” BMJ Quality & Safety 25, no. 7 (2016): 509–517, 10.1136/BMJQS-2015-004315. PMC4941163 26376674

[6] J. Hummel and P. Evans , Providing Clinical Summaries to Patients After Each Office Visit: A Technical Guide (Seattle: Qualis Health. Published online, 2012).

[7] C. Blease , J. Walker , C. M. DesRoches , and T. Delbanco , “New U.S. Law Mandates Access to Clinical Notes: Implications for Patients and Clinicians,” Annals of Internal Medicine 174, no. 1 (2021): 101–102, 10.7326/M20-5370. 33045176

[8] S. Pathak , G. Summerville , C. P. Kaplan , S. S. Nouri , and L. S. Karliner , “Patient‐Reported Use of the After Visit Summary in a Primary Care Internal Medicine Practice,” Journal of Patient Experience 7, no. 5 (2020): 703–707, 10.1177/2374373519879286. 33294604 PMC7705830

[9] K. Turner , A. Clary , Y. R. Hong , A. Alishahi Tabriz , and C. M. Shea , “Patient Portal Barriers and Group Differences: Cross‐Sectional National Survey Study,” Journal of Medical Internet Research 22, no. 9 (2020): e18870, 10.2196/18870. https://www.jmir.org/2020/9/e18870. 32940620 PMC7530687

[10] N. Couët , S. Desroches , H. Robitaille , et al., “Assessments of the Extent to Which Health‐Care Providers Involve Patients in Decision Making: A Systematic Review of Studies Using the Option Instrument,” Health Expectations 18 (2013): 542–561, 10.1111/hex.12054. 23451939 PMC5060794

[11] D. L. Roter , “Physician/Patient Communication: Transmission of Information and Patient Effects,” Maryland State Medical Journal 32, no. 4 (1983): 260–265. 6865482

[12] D. J. Kiesler and S. M. Auerbach , “Optimal Matches of Patient Preferences for Information, Decision‐Making and Interpersonal Behavior: Evidence, Models and Interventions,” Patient Education and Counseling 61, no. 3 (2006): 319–341, 10.1016/j.pec.2005.08.002. 16368220

[13] M. A. Clarke , J. L. Moore , L. M. Steege , et al., “Health Information Needs, Sources, and Barriers of Primary Care Patients to Achieve Patient‐Centered Care: A Literature Review,” Health Informatics Journal 22, no. 4 (2016): 992–1016, 10.1177/1460458215602939. 26377952

[14] A. Tong , P. Sainsbury , and J. Craig , “Consolidated Criteria for Reporting Qualitative Research (COREQ): A 32‐item Checklist for Interviews and Focus Groups,” International Journal for Quality in Health Care 19, no. 6 (2007): 349–357, 10.1093/intqhc/mzm042. 17872937

[15] G. Guest , A. Bunce , and L. Johnson , “How Many Interviews Are Enough? An Experiment With Data Saturation and Variability,” Field Methods 18, no. 1 (2006): 59–82, 10.1177/1525822X05279903.

[16] H. F. Hsieh and S. E. Shannon , “Three Approaches to Qualitative Content Analysis,” Qualitative Health Research 15, no. 9 (2005): 1277–1288, 10.1177/1049732305276687. 16204405

[17] P. Barr , M. Dannenberg , C. Ganoe , et al. A Case Study of U.S. Clinics that Routinely Offer Patients Recordings of Clinic Visits. In: 10th International Shared Decision Making Conference; 2019.

[18] P. C. Tang , C. Newcomb , S. Gorden , and N. Kreider Meeting the Information Needs of Patients: Results From a Patient Focus Group. Proceedings of the AMIA Annual Fall Symposium (1997, 4 Suppl.), S672. PMC2233596 9357710

[19] P. C. Tang , M. A. Jaworski , C. A. Fellencer , N. Kreider , M. P. LaRosa , and W. C. Marquardt Clinician Information Activities in Diverse Ambulatory Care Practices. Proceedings of the AMIA Annual Fall Symposium . Published online 1996:12. PMC2232980 8947618

[20] G. Deledda , F. Moretti , M. Rimondini , and C. Zimmermann , “How Patients Want Their Doctor to Communicate: A Literature Review on Primary Care Patients' Perspective,” Patient Education and Counseling 90, no. 3 (2013): 297–306, 10.1016/J.PEC.2012.05.005. 22709720

[21] M. A. Clarke , L. M. Steege , J. L. Moore , R. J. Koopman , J. L. Belden , and M. S. Kim , “Determining Primary Care Physician Information Needs to Inform Ambulatory Visit Note Display,” Applied Clinical Informatics 5, no. 1 (2014): 169–190, 10.4338/ACI-2013-08-RA-0064. 24734131 PMC3974234

[22] P. Edelman , D. Kuhn , B. R. Fulton , and G. A. Kyrouac , “Information and Service Needs of Persons With Alzheimer's Disease and Their Family Caregivers Living in Rural Communities,” American Journal of Alzheimer's Disease & Other Dementias® 21, no. 4 (2006): 226–233. 10.1177/1533317506290664 PMC10833300 16948286

[23] J. M. Clayton , P. N. Butow , and M. H. N. Tattersall , “The Needs of Terminally Ill Cancer Patients Versus Those of Caregivers for Information Regarding Prognosis and End‐of‐Life Issues,” Cancer 103, no. 9 (2005): 1957–1964, 10.1002/cncr.21010. 15789363

[24] R. W. R. Bratches , N. Z. Freundlich , J. N. Dionne‐Odom , A. J. O'Malley , and P. J. Barr , “Perceptions of the Impact of COVID‐19 on Healthcare Communication in a Nationally Representative Cross‐Sectional Survey of Family Caregivers,” BMJ Open 12, no. 4 (2022): e051154, 10.1136/bmjopen-2021-051154. PMC9016173 35418422

[25] I. Hargraves , A. LeBlanc , N. D. Shah , and V. M. Montori , “Shared Decision Making: The Need For Patient‐Clinician Conversation, Not Just Information,” Health Affairs 35, no. 4 (2016): 627–629, 10.1377/HLTHAFF.2015.1354. 27044962

[26] S. Weiner , A. Schwartz , L. Altman , et al., “Evaluation of a Patient‐Collected Audio Audit and Feedback Quality Improvement Program on Clinician Attention to Patient Life Context and Health Care Costs in the Veterans Affairs Health Care System,” JAMA Network Open 3, no. 7 (2020): e209644, 10.1001/JAMANETWORKOPEN.2020.9644. 32735338 PMC7395234

